# Perfusion Decellularization of Extrahepatic Bile Duct Allows Tissue-Engineered Scaffold Generation by Preserving Matrix Architecture and Cytocompatibility

**DOI:** 10.3390/ma14113099

**Published:** 2021-06-05

**Authors:** Yolik Ramírez-Marín, David Eduardo Abad-Contreras, Martha Ustarroz-Cano, Norma S. Pérez-Gallardo, Lorena Villafuerte-García, Dulce Maria Puente-Guzmán, Jorge Luna del Villar-Velasco, Leonardo Alejandro Rodríguez-López, Gonzalo Torres-Villalobos, Miguel Ángel Mercado, Jesús Tapia-Jurado, Francisco Drusso Martínez-García, Martin Conrad Harmsen, M. Cristina Piña-Barba, David M. Giraldo-Gomez

**Affiliations:** 1 Program of Medical Specialization General Surgery, Division of Posgraduate Studies, Faculty of Medicine, National Autonomous University of Mexico (UNAM), Avenida Universidad 3000, Circuito de Posgrados, Unidad de Posgrado Edificio “E” 2° piso, Ciudad Universitaria, Coyoacán, Ciudad de México 04510, Mexico; jorgeramy@gmail.com; 2 National Institute of Medical Sciences and Nutrition of Mexico Salvador Zubirán, Vasco de Quiroga 15, Belisario Domínguez Secc. 16, Tlalpan, Ciudad de México 14080, Mexico; akumakuja@hotmail.com (L.A.R.-L.); gonzalo.torresv@incmnsz.mx (G.T.-V.); miguel.mercadod@incmnsz.mx (M.Á.M.); 3 Laboratory for Biomaterials, Materials Research Institute, National Autonomous University of Mexico (UNAM), Avenida Universidad 3000, Circuito Exterior, Ciudad Universitaria, Coyoacán, Ciudad de México 04510, Mexico; deabad91@gmail.com (D.E.A.-C.); crispina99@gmail.com (M.C.P.-B.); 4 Department of Cell and Tissue Biology, Faculty of Medicine, National Autonomous University of Mexico (UNAM), Avenida Universidad 3000, Circuito Interior, Edificio “A” 3° piso, Ciudad Universitaria, Coyoacán, Ciudad de México 04510, Mexico; ustarrozcano@hotmail.com; 5 Surgical Training Section, Faculty of Veterinary Medicine and Animal Husbandry, National Autonomous University of Mexico (UNAM), Avenida Universidad 3000, Circuito Exterior, Ciudad Universitaria, Coyoacán, Ciudad de México 04510, Mexico; perezgallardo55@gmail.com (N.S.P.-G.); mvz.villafuerte@gmail.com (L.V.-G.); dulcempgmvz@gmail.com (D.M.P.-G.); jorgevelasco1259@gmail.com (J.L.d.V.-V.); 6 Unit of Advanced Medical Simulation, Division of Posgraduate Studies, Faculty of Medicine, National Autonomous University of Mexico (UNAM), Avenida Universidad 3000, Circuito de Posgrados, Unidad de Posgrado Edificio “B” 2° piso, Ciudad Universitaria, Coyoacán, Ciudad de México 04510, Mexico; tapiajj@amcg.org.mx; 7 Department of Pathology and Medical Biology, University Medical Center Groningen University of Groningen, Hanzeplein 1, 9713 Groningen, The Netherlands; f.d.martinez.garcia@umcg.nl (F.D.M.-G.); m.c.harmsen@umcg.nl (M.C.H.); 8 Microscopy Core Facility, Faculty of Medicine, National Autonomous University of Mexico (UNAM), Avenida Universidad 3000, Circuito Interior, Edificio “A” planta baja, Ciudad Universitaria, Coyoacán, Ciudad de México 04510, Mexico

**Keywords:** decellularization, bile duct, matrix scaffold, extracellular matrix (ECM)

## Abstract

Reconstruction of bile ducts damaged remains a vexing medical problem. Surgeons have few options when it comes to a long segment reconstruction of the bile duct. Biological scaffolds of decellularized biliary origin may offer an approach to support the replace of bile ducts. Our objective was to obtain an extracellular matrix scaffold derived from porcine extrahepatic bile ducts (dECM-BD) and to analyze its biological and biochemical properties. The efficiency of the tailored perfusion decellularization process was assessed through histology stainings. Results from 4’-6-diamidino-2-phenylindole (DAPI), Hematoxylin and Eosin (H&E) stainings, and deoxyribonucleic acid (DNA) quantification showed proper extracellular matrix (ECM) decellularization with an effectiveness of 98%. Immunohistochemistry results indicate an effective decrease in immunogenic marker as human leukocyte antigens (HLA-A) and Cytokeratin 7 (CK7) proteins. The ECM of the bile duct was preserved according to Masson and Herovici stainings. Data derived from scanning electron microscopy (SEM) and thermogravimetric analysis (TGA) showed the preservation of the dECM-BD hierarchical structures. Cytotoxicity of dECM-BD was null, with cells able to infiltrate the scaffold. In this work, we standardized a decellularization method that allows one to obtain a natural bile duct scaffold with hierarchical ultrastructure preservation and adequate cytocompatibility.

## 1. Introduction

Cholecystectomy or gall bladder removal is an intraabdominal surgical procedure with 750,000 patients treated annually, solely in the United States. These patients generally develop life-threatening iatrogenic-induced bile duct injuries [1,2,3], which require surgical reconstruction procedures like Roux-en-Y hepaticojejunostomy [4,5]. This is a major surgical procedure that frequently causes complications such as stenosis, recurrent cholangitis, and secondary biliary cirrhosis [6]. Hence, novel therapeutic options are needed that aim to preserve the anatomy and physiology of the bile duct region.

Decellularization is a process that generates scaffolds of natural origin, with the aim to replace or repair diseased organs or tissues. During the decellularization process, most of the tissular cellular antigens that may elicit an immune response in the host body should be removed [7]. As antigens are removed, this technique may generate acellular scaffolds from both allogeneic or xenogeneic sources, thus overcoming current organ donor shortages. Ideally, the decellularization process must preserve native tissue extracellular matrix (ECM) components, many of which are necessary for cell survival, proliferation, and differentiation [8]. Acellular scaffolds derived from healthy bile ducts of animal sources (e.g., porcine origin) may provide a low-cost and widely accessible off-the-shelf replacements [9]. The use of decellularized matrices for bile duct repair has focused on employing tubular tissues distinct from bile duct, such as ureter [9]. Furthermore, the animal sources employed in decellularization vary greatly and lack human anatomy resemblance [9,10,11,12]. Therefore, using porcine decellularized extrahepatic bile duct scaffolds may tackle the disadvantages before-mentioned, due to the anatomical similarities between species.

Based on previously reported decellularization protocols [9,13], in this study, we developed a protocol to generate a decellularized extracellular matrix from pig bile ducts (dECM-BD). In this work, we optimized the before-mentioned protocols, replacing commonly used decellularization reagents such as triton and sodium deoxycholate with others such as sodium dodecyl sulfate (SDS) and deoxyribonuclease (DNase) enzyme. We hypothesized that replacing such reagents could lead to an adequate preservation of native ECM integrity. Additionally, by avoiding triton and sodium deoxycholate during decellularization, we could prevent any potential leachable that could hamper cell viability in vitro and prevent related cytotoxicity concerns before patient implantation. Therefore, in this work, we describe a method for porcine bile duct decellularization, going from tissue harvesting to evaluation of the microarchitecture, and demonstrating the biological properties of dECM-BD scaffolds aimed at tissue engineering.

## 2. Materials and Methods

### 2.1. Ethical Guidelines

All experiments involving animal use were conducted following the current Mexican animal welfare act, NOM-062-ZOO-1999, and the guidelines of the EU Directive 2010/63/EU for animal experiments [14,15]. Furthermore, the animal use protocol was evaluated and approved by the Ethics Committee of the Faculty of Medicine from the Universidad Nacional Autónoma de México (UNAM), project number FM/DU085/2017.

### 2.2. Bile Duct Harvest

**Figure 1:**
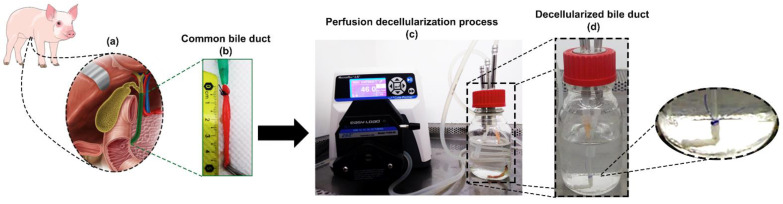
Surgical process and decellularization process. Traction of the fundus of the gallbladder and a reference of the porcine biliary tract was conducted with the previous enterotomy of the second portion of the duodenum and the splinting of the Vater’s ampulla. (**a**). Extrahepatic native biliary tract (**b**). Perfusion of the bile duct in a closed circuit by a peristaltic pump (**c**). Images depict change from native (**c**), resulting decellularized bile duct appeared white (**d**).

For these experiments, a total of 9 young Landrace male pigs (weight 25–30 kg) were employed. All animals were euthanized by anesthesia overdose (sodium pentobarbital). Under aseptic surgical conditions, the abdominal cavity was accessed by mid-line incision laparotomy. The bile duct was visualized by relocating the liver from its normal anatomical position (Figure 1a).

Both gallbladder and cystic gallbladder were used as anatomical reference points. Subsequently, enterotomy of the second portion of the duodenum was performed to locate the Vater’s ampulla, which was then cannulated using an 8 Fr catheter (Figure 1b).

The extrahepatic bile duct was dissected from the duodenum down to its entrance into the liver. Any surrounding connective tissue was manually removed. Approximately 5 to 8 cm of biliary duct tissue was obtained per procedure. The bile ducts were rinsed with phosphate buffered saline (PBS) solution after surgical removal and stored in this solution at 4 °C until subsequent decellularization (Figure 1c).

### 2.3. Decellularization Protocol

The selection of decellularization reagents was based on modifying preexistent protocols presented by Giraldo et al. and Cheng et al. [9,13], briefly described as follows: All solutions were perfused via the bile duct lumen connecting each bile duct to a caterer number 14 attached by a 2-0 silk suture. The process started with perfusion and recirculation of 1% SDS for 96 h, with changes every 24 h. Then, PBS (1X) was employed for an additional 24 h to remove any remains of SDS from the tissue. After this, samples were perfused by DNase-I solution (10mg/mL) in a Dulbecco’s Phosphate Buffered Saline (DPBS) solution, which contains calcium and magnesium (Gibco, Thermo Fisher Scientific, Waltham, MA, USA), for 24 h. Lastly, Milli-Q water was perfused for 24 h to remove any potential cell-debris present (Figure 1c,d).

A closed-circuit peristaltic pump (Masterflex L/S Standard Digital Pump, Antylia Scientific, Vernon Hills, IL, USA) was used during all processes. The flow rate was of 46.0 mL/min at room temperature (23 °C) (Figure 1c). The volume of the decellularization solutions in the organ reservoir was 200 mL. The decellularization protocol took eight days in total.

### 2.4. Mesurement of Decellularization

To estimate the degree of decellularization achieved in our process, DNA quantification, histological analysis, immunohistochemical analysis, nuclei quantification, and positive stain area quantification of immunohistochemical images were done.

#### 2.4.1. DNA Quantification

Specimens (n = 9) from each sample of 15 mg were homogenized in 1 mL of denaturing solution using a TISSUE LYSER (Qiagen Inc. Germantown, MD, USA) to quantify the total DNA content of both native samples and decellularized matrices (4 cycles at 10000 rpm for 2 min).

The DNA content was isolated with TRIzol (Invitrogen, Thermo Fisher Scientific, Waltham, MA, USA), and the concentration was quantified by the DNA HS assay kit in Qubit™ 3.0 Fluorometer (Invitrogen, Thermo Fisher Scientific, Waltham, MA, USA) according to the manufacturer’s instructions. The result of DNA quantification was normalized by the weight of wet tissue.

#### 2.4.2. Histological Analyses

Histological analyses were performed on native tissue samples and decellularized matrices. For processing, the samples were gradually dehydrated from 70% to 99.99% ethanol. Tissue samples were fixed in Xylene (Merck, Darmstadt, Germany) and embedded in paraffin (Sigma-Aldrich, Merck, Darmstadt, Germany). Sections of 5 μm were produced using a rotary microtome (RM2125 RTS, Leica, Buffalo Grove, IL, USA). Finally, the sections were stained with 4’-6-diamidino-2-phenylindole (DAPI) (Abcam, Cambridge, UK), hematoxylin and eosin (Merck, Darmstadt, Germany), Masson’s Trichrome (Merck, Darmstadt, Germany), and Herovici (Merck, Darmstadt, Germany) protocols as previously reported [16,17].

#### 2.4.3. Immunohistochemical Analyses

Immunohistochemical analyses were performed on native tissue samples and decellularized matrices. Sectioned samples (5 μm) were processed for conventional immunohistochemistry to determine the presence of Cytokeratin 7 and HLA-A in the MEC. Sections were mounted on positive-charged slides (Shandon Inc., Pittsburgh, PA, USA), dewaxed with xylene, rehydrated with PBS, and then transferred to plastic Coplin jars containing Diva Decloaker (DV2004, Biocare, Concord, CA, USA), for antigen retrieval. The slides inside Coplin jars were pressure heated for 20 min at ∼60 °C, followed by 3 min at ∼110 °C. The slides were cooled in a jar at room temperature for 15 min and then soaked in PBS. Endogenous peroxidase was blocked with 30% H_2_O_2_ for 10 min, followed by three PBS rinses.

The samples were then incubated for 10 min at room temperature with Background Sniper (protein blocker) ready-to-use solution (BS966 Biocare, Concord, CA, USA); the protein blocker was drained. Sections were incubated with 1:100 dilution of anti-Cytokeratin 7 antibody (sc-23876, Santa Cruz Biotechnology, Inc. OR, USA.) and 1:100 dilution of recombinant anti-HLA-A antibody (ab52922, Abcam, Cambridge, UK) in PBS humidified chamber overnight at 4 °C. The following day, all the sections were rinsed three times with PBS and then incubated for 20 min at room temperature with the secondary biotinylated antibody. The avidin-biotin-HRP complex was used, and the reaction was developed with 3′3′ diaminobenzidine according to the supplier’s instructions (all reagents were from Starr Trek Universal HRP Detection System, 901-STUHRP700, BioCare, Concord, CA, USA). Slides were covered with synthetic mounting resin and observed in an Axio Zoom.V16 microscope (Zeiss, Oberkochen, Germany).

#### 2.4.4. Nuclei Quantification

The number of positive nuclei from cells was labeled with DAPI. For each sample, five representative areas were selected using 40× magnification images acquired with Axio Zoom.V16 microscope (Zeiss, Oberkochen, Germany). The acquired images were transformed into 8-bit with ImageJ software (Java 1.8.0_172), and the background was subtracted with 5 pixels and marking a lower limit of 20 and an upper limit of 255. Finally, the particle count was made from 104.23 to infinity in squared pixels. The number of DAPI positive particles were counted in ImageJ (RRID:SCR_003070).

#### 2.4.5. Positive Stain Area Quantification of Immunohistochemical Images

Quantification of CK7 and HLA-A positive stain was conducted by the software Dragonfly 4.1 (Object Research System, Montreal, QC, Canada), and for native and decellularized samples five representative areas were selected used a 40x magnification images. The images were converted to RGB, and the channel positive to the stain was selected to carry out the quantification area. The segmentation process was done via multi-ROI tool of the software by define range. The painting tool of the positive stain area in the multi-ROI was matched with the original images, and finally the area was measured selecting surface area from the multi-ROI.

### 2.5. Characterization of the Decellularized Extracellular Matrix Scaffold Derived from Porcine Extrahepatic Bile Ducts (dECM-BD)

#### 2.5.1. Microarchitectural Imaging

The microarchitecture of the native samples and dECM-BD was observed by SEM. Briefly, all samples were fixed in 3% (*v*/*v*) glutaraldehyde in a buffering solution of 0.1M sodium cacodylate (pH 7.2) for 48 h, (16538, Electron Microscopy Science, Hatfield, PA, USA). Subsequently, all samples were dehydrated at various ethanol concentrations, ranging from 30% to 99.99%, 30 min each, (E7148, Sigma-Aldrich, Merck, Darmstadt, Germany) Finally, they were dried using a critical point dryer CO_2_ chamber (K850, Quorum Technologies, Kent, UK), as previously reported [18,19]. The images were acquired with a FIB-SEM Crossbeam 550 field emission microscope (Zeiss Oberkochen, Germany) at 5 kV.

#### 2.5.2. Thermogravimetric Analysis

Tissue samples underwent thermogravimetric analysis (TGA) to assess the thermal stability and to find any matrix degradation due to the decellularization protocol. For TGA, both native and decellularized samples were freeze-dried at 3.6 Pa and −47 °C in a Freeze Dryer (FreeZone 1, Labconco, Kansas City, MO, USA). The experiment was carried out in a thermogravimetric analyzer (Q5000, TA Instruments, New Castle, DE, USA) with a heating rate of 10 °C/min under a nitrogen atmosphere with a temperature range from 32 °C to 500 °C (from near human body normal temperature to elevated temperatures to characterization purposes of the proteins components of the biomaterial).

### 2.6. Cytocompatibility of dECM-BD

#### 2.6.1. Detection of Leachable Components and Cytotoxicity in dECM-BD

The presence of leachable components and dECM-BD cytotoxicity was measured by the method described by Getova V (2019) [20]. Briefly, dECM-BD was incubated at 37 °C in a (1×) PBS containing 1% Penicillin-Streptomycin (*v*/*v*) for 72 h. The dECM-BD was washed twice with PBS and incubated a second time in high-glucose Dulbecco’s Modified Eagle Medium (DMEM) containing 10% FBS (*v*/*v*), 1% Pen-Streptomycin (*v*/*v*), and L-Glutamine for 72 h in static conditions. Human dermal fibroblasts (PK84) were seeded in 96 well plates at 10,000 cells/cm² seeding density and left to attach at 37 °C, 5% CO_2_ for two hours. The medium was removed, and the cells exposed at this point on to the dECM-BD conditioned medium (CMe) containing any potential leachable using a twofold dilution series. Positive controls to induce cell toxicity were determined with two concentrations of puromycin (Gibco, Thermo Fisher Scientific, Waltham, MA, USA): 10 mg/mL and 5 mg/mL. Following 7 days, cytotoxicity was determined using MTT (3–(4,5-dimethylthiazol-2-yl)-2,5- diphenyltetrazolium bromide) colorimetric assay (5 mg/mL). The well plates were incubated for 3 h at 37 °C. Afterwards, the media was removed, and purple formazan crystals dissolved in 200 µL of dimethyl sulfoxide (DMSO) per well. Optical density was measured at 585 nm and 650 nm. The subtraction values of OD585 nm and OD650 nm were plotted against the log dilution to determine the half-maximal inhibitory concentration (IC50). The IC50 is a dose-response curve that determines the dose needed to induce cell death in 50% of a population. Analyses of the IC50 were carried out in Graph Pad Prism 6.05 using a nonlinear regression for the dose-response inhibition curve.

#### 2.6.2. Cell Infiltration into dECM-BD

Despite the care taken in obtaining dECM-BD, cytotoxic traces may remain, so it is necessary to evaluate the ability of dECM-BD to be repopulated with human cells through its infiltration, for which human fibroblasts were used (PK84) (3000 cells per cm^3^). The cells were seeded on the dECM-BD after passage 2 (P2) using high-glucose Dulbecco’s Modified Eagle Medium (DMEM) containing 10% FBS (*v*/*v*), 1% Pen-Streptomycin (*v*/*v*), and L-Glutamine (all from Gibco, Thermo Fisher Scientific, Waltham, MA, USA), and the constructs were incubated for seven days at 37 °C, 5% CO_2_ in static conditions. Afterward, the samples were washed twice with PBS and fixed in 4% of paraformaldehyde and evaluated with Masson’s Trichrome staining [17].

### 2.7. Statistical Analysis

Statistical analysis was performed using GraphPad Prism 6.05 statistical software (San Diego, CA, USA). The decellularized group was compared with a native sample group as a control using a *t*-test mean comparison. A *p*-value of ˂0.05 was considered significant, n = 9.

## 3. Results

### 3.1. Decellularization Assessment

**Figure 2:**
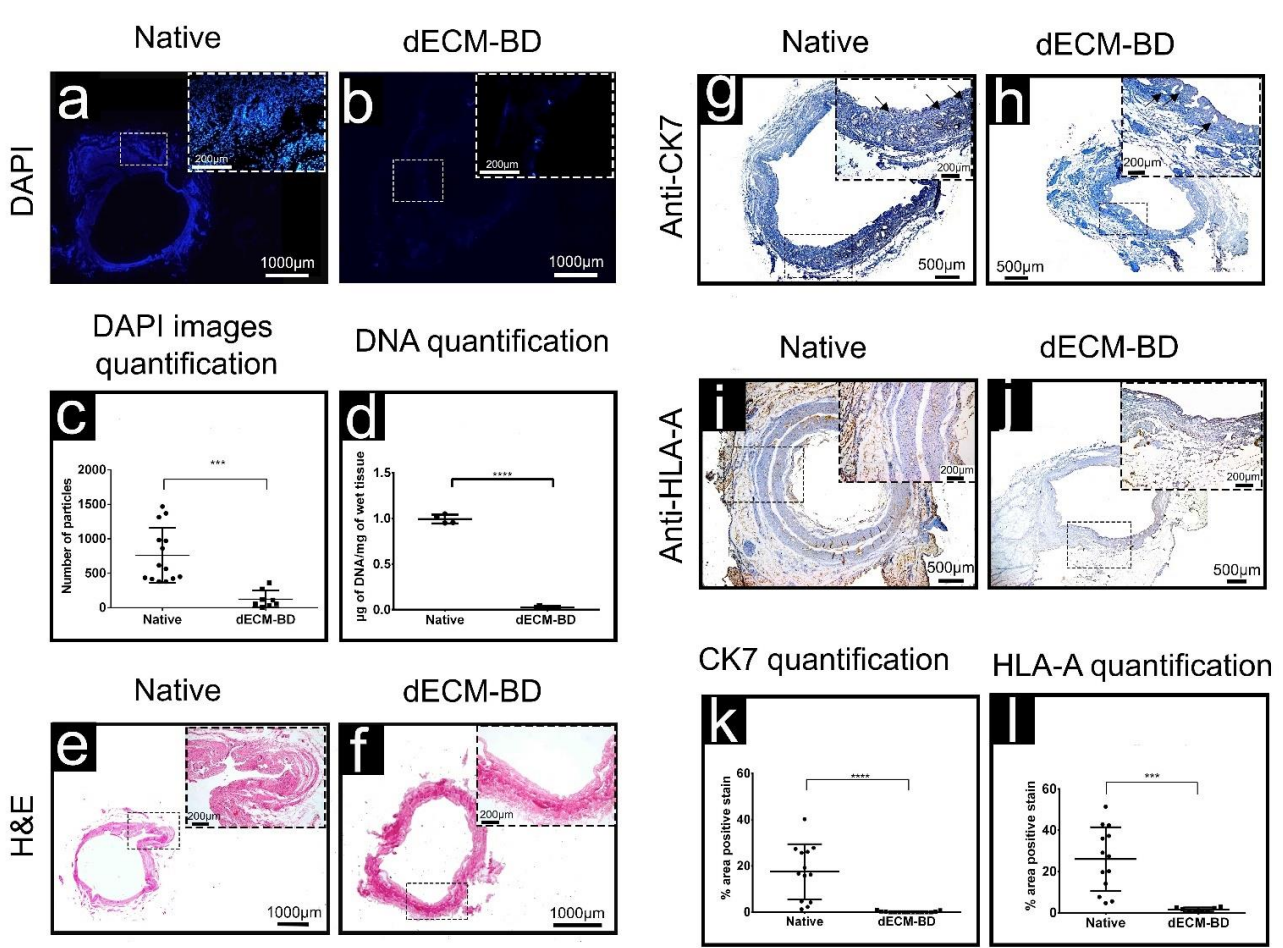
Decellularization assessment. The spatial DAPI nuclei labeling comparison of native bile duct (**a**) and decellularized (dECM-BD) (**b**). Quantification of DAPI images showed a significant decrease of positively stained particles (i.e., nuclei) in the dECM-BD samples (**c**). The quantification of residual DNA confirmed the decrease of DNA content in dECM-BD samples, and **** *p* < 0.05 is considered significant. Data are expressed as the mean ±SD (n = 9) (**d**). H&E stains confirmed the removal of cells (**e**,**f**). The comparison of the native and decellularized bile ducts by immunohistochemistry of anti-CK7 (**g**,**h**), and anti-human leukocyte antigens (anti-HLA-A) (**i**,**j**) showed the decrease of these cells’ proteins in the decellularized samples (**h**,**j**). The positive labeling of CK7 cell protein is observed in the submucosa region of the native bile duct (**g**), and the dECM-BD show the elimination of CK7 (**h**). The arrows indicate the lumen conservation of the vascular plexus after the decellularization process (**g**,**h**). Quantification of anti-CK7 (**k**) and anti-HLA-A (**l**) from the immunohistochemistry images showing a significant difference between native and dECM-BD; **** *p* < 0.05 and *** *p* < 0.05 were considered significant from CK7 and HLA-A, respectively. Data are expressed as the mean ±SD (n = 9).

Nuclei labeling with DAPI showed an effective decrease in the number of positively stained particles after the completion of decellularization process (Figure 2a,b). The presence of fluorescence after decellularization in DAPI images (Figure 2b) is partially explained by the autofluorescence from collagen in the samples [21]. Additionally, our assessment showed a 2% residual DNA. While this proportion is below the safety requirements established by (DNA < 3 ug/mL), DAPI would still be able to bind to any residual DNA, to AT (adenine-thymine) complexes, giving a positive DAPI staining in decellularized tissue. Quantification of DAPI stained nuclei confirmed a significant decrease in the number of nuclei after decellularization from 760.1 ± 106.6 nuclei (native) to 126.9 ± 44.1 nuclei (dECM-BD) (Figure 2c). Additionally, quantification of DNA also showed a 98% decrease of DNA after decellularization, from 0.993 ± 0.024 µg/mg (native) to 0.027 ± 0.007 µg/mg (dECM-BD) (Figure 2d). H&E staining also confirmed a decrease in the number of stained nuclei (Figure 2e,f).

As additional assessment of the decellularization process, immunostaining of HLA-A and CK7 positive cells confirmed a decrease of both proteins in the dECM-BD when compared to native tissue (Figure 2g–j). Furthermore, dECM-BD retained the lumen structures of blood vessels in the vascular plexus, marked by arrows (Figure 2g,h). Quantification of CK7 mean values were 17.55 ± 3.31% and 0.18 ± 0.08% for native tissue and dECM-BD, respectively. For HLA-A, mean values were 26.04 ± 4.28% and 1.60 ± 0.36% for native and dECM-BD samples, respectively (Figure 2k,l, respectively).

### 3.2. ECM Assessment

**Figure 3:**
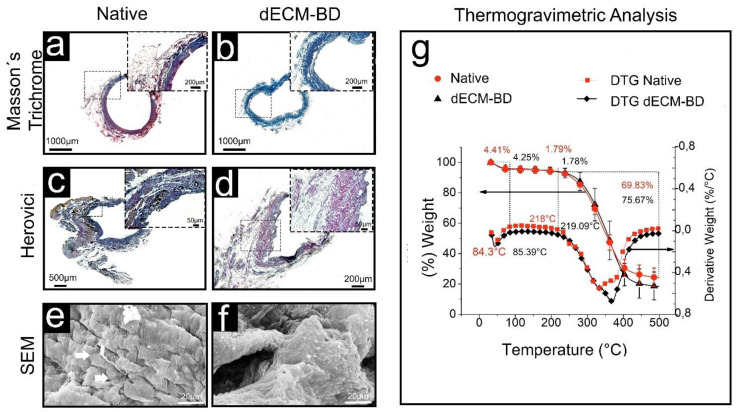
Characterization of the dECM-BD. Masson’s trichrome stain of native (**a**) and decellularized bile duct (dECM-BD) (**b**), Herovici stain of native ECM (**c**), and dECM-BD (**d**). Scanning electron microscopy (SEM) microphotographs of dECM-BD samples and native. The epithelium is observed in the sample from the bile duct with primary cilia indicated by the white arrows (**e**). Decellularized sample in which cellular material is absent in pores, and microvilli, with evidence of conserved extracellular matrix (**f**). Thermogravimetric analysis (TGA) results from native and decellularized samples. DTG: derivate thermogravimetric (**g**).

Macroscopically, a gradual loss of red color leaving a white duct was observed during the perfusion decellularization process (Figure 1c,d). The decellularization process generated a dECM-BD that retained its gross anatomical structure. Masson’s trichrome and Herovici stains were performed to evaluate the microarchitecture relative to the presence of major native tissue ECM components (Figure 3a–d). In native bile ducts, the collagen (blue) and cellular components such as the cytoplasm and muscle layers (red) are stained (Figure 3a). After decellularization, only blue-stained structures were visible (Figure 3b,d). The Herovici stain distinguished between mature (red) and immature (blue) collagen (Figure 3c) [22]. Hence, Herovici staining indicated that crosslinked or packed/mature collagen was the major component retained after decellularization (Figure 3d).

### 3.3. Microarchitectural and Thermal Integrity of Decellularized Matrices

Microstructural changes were observed in dECM-BD samples respect to native ECM by SEM. Native tissue showed a typical arrangement, with the apical surfaces of cholangiocytes with cilium (marked by white arrows), as well as pores (Figure 3e). In dECM-BD samples, no cells, microvilli, or cilium were visible. The pores of the ECM and the collagen fibers in the decellularized samples were preserved (Figure 3f).

The TGA profiles (Figure 3g) of native and dECM-BD samples were typical of collagen (the main structural component of the ECM) [13]. There was no significant difference between the samples. These curves show three weight losses, and the temperature of the thermal transitions was located with the derivate thermogravimetry (DTG) curve where an inflexion was observed at the thermal transition. The first weight loss of 4.41% was observed up to 84.3 °C for native samples and 4.25% up to 85.39 °C for dECM-BD samples. The second weight loss observed was 1.79% up to 218 °C for native samples and 1.78% up to 219.09 °C for dECM-BD. Finally, the most substantial weight loss of 69.83% occurred up to 500 °C for native sample and 75.67% for dECM-BD up to the same temperature.

### 3.4. Cytotoxicity

**Figure 4:**
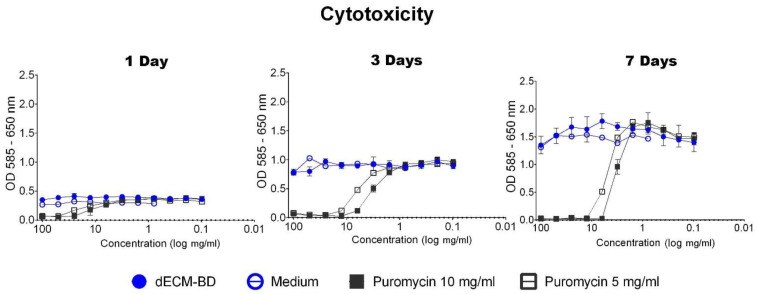
Cytotoxicity evaluation of the dECM-BD. Optical density plotted against the serially diluted conditioned medium (CMe) from decellularized extracellular matrix bile ducts (dECM-BD) at three different time points (log scale). PK84 fibroblasts were treated with dECM-BD CMe, cell culture media (negative control for cell death), and two different concentrations of puromycin, to induce a positive cytotoxic response. Results are presented as mean with a standard error of the mean of triplicates of three independent donors.

PK84 cells exposed to dECM-BD CMe showed no difference in MTT conversion at any time point when compared to cells exposed to regular cell culture medium. Therefore, no IC50 was calculated, indicating that the dECM-BD did not release detectable cytotoxic compounds over time (Figure 4). Results show that puromycin induced strong cytotoxic response at 10 mg/mL and 5 mg/mL in all timepoints. The LogIC50 of puromycin at day 1 was 4.46 and 3.41; at day 3 it was 2.63 and 5.66; and at day 7 it was 3.17 and 5.54 for 10 mg/mL and 5 mg/mL concentrations, respectively (Figure 4).

### 3.5. Cell Infiltration into dECM-BD

**Figure 5:**
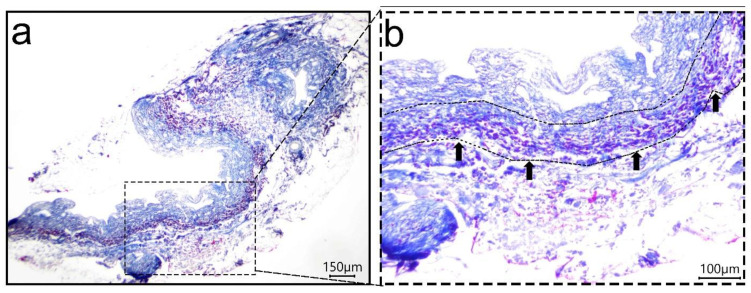
Cell infiltration of the dECM-BD 7 days post seeding. Masson’s trichrome stain after 7 days of cell seeding in the dECM-BD (**a**), and the arrows in the magnification show the infiltration of cells in the decellularized matrix (**b**). (Luminal side is in the upper side of the images).

Infiltration of cells into the dECM-BD after 7 days in culture under static conditions was confirmed by Masson’s trichrome staining. Presence of cells was observed in the submucosa of dECM-BD, shown with arrows in Figure 5.

## 4. Discussion

In our current research, we describe a novel decellularization protocol based on detergent-enzymatic perfusion for extrahepatic bile duct tissues of porcine origin. Our protocol demonstrated not only successful decellularization but also architectural preservation of the extracellular matrix (ECM). Furthermore, the decellularization process and the so-called dECM-BD obtained was not cytotoxic and allowed for cell infiltration after a week in static culture conditions.

The production of acellular matrices from bile ducts by decellularization offers a promising alternative approach for functional tissue replacement. The aims for successful decellularization methods are first the effective removal of cellular material that can lead to an immune response and second the preservation of ECM structure [23,24]. Effective decellularization is necessary to prevent immune and inflammatory reactions. However, the efficiency of a given decellularization method or protocol depends on the characteristics of the tissue of interest [7]. The proposed protocol allows for the decellularization of bile ducts while preserving the ECM integrity and architecture, comparable to that of native tissue (Figure 1b–d). The resulting dECM-BD gathers the rigorous requirement to define successful decellularization, the absence of nuclear material as shown with DAPI staining (Figure 2a,b), and DNA < 0.03 µg/mg of ECM weight as demonstrated quantitatively (Figure 2d) [25]. This requirement is critical because residual DNA fragments in dECM-BD have shown to lead to cytotoxicity in vitro and adverse immunological response after implantation in animal models [26,27,28].

The immunohistochemistry results showed the labeling of CK7 in the mucosa native samples (Figure 2g), which is consistent with similar studies [29], and showed no CK7 positive cells of biliary mucosa in the decellularized samples (Figure 2h). CK7 is a key biliary epithelial cells marker [29]; thus, its absence n dECM-BD samples confirmed the effectiveness of the decellularization process. We also showed that native bile duct labeled positively for HLA-A (Figure 2i) [30,31]. In contrast, the dECM-BD revealed less than 1.7% to mark positively for both CK7 and HLA-A in the submucosal glandular components (Figure 2k,l). This result is our first evidence to suggest that the dECM-BD may be non-immunogenic. However, further studies are necessary to expand on these findings.

Another essential consideration for tissue decellularization is to minimize the undesirable alteration and loss of ECM components [7,25]. Our dECM-BD demonstrated maintenance of the critical structural ECM components like collagen, as shown in the Masson staining, where blue coloration remained after the decellularization process (Figure 3a,b). This was confirmed by the Herovici staining images (Figure 3c,d), where the coloration obtained is between blue and red color according to the density of the collagen in the sample; blue stains young or less densely packed collagen, while mature or highdensely packed collagen stains red and results in a combination that provides the color according to the density of collagen present in the tissue [22]. Hence, the major remaining component in the dECM-BD is dense or more packing collagen. The maintenance of the hierarchical structure is critical for in vivo performance of decellularized grafts, as shown in previous results of ureteral graft used for bile duct reconstruction in swine models [9].

The dECM-BD preserved the structure of the peribiliary vascular plexus, connective tissue, mucosa, and submucosa (Figure 2 and Figure 3) [25]. SEM data corroborated these findings in regard to porosity and 3D microarchitecture (Figure 3e,f), between native and decellularized tissue. Moreover, the dECM-BD obtained with the current protocol preserved the architecture of the vessel lumen, comparable to native bile ducts (Figure 2). Inadequate blood supply from the hepatic artery and perivascular plexus can lead to biliary duct ischemic cholangiopathy [32,33,34,35]. Such ischemic event constitutes one of the most common complications following hepato-pancreatic-biliary surgical procedures [35,36]. Consequently, oxygen and nutrient supply via an vascularized stroma is essential for the long-term survival of bioengineered bile ducts. Further studies will aim to the recellularization of the architectural preserved dECM-BD vascular network. The TGA of the ECM’s structural integrity (Figure 3g) of the evaluated samples showed a typical curve for collagen, which agrees with previous studies [37,38,39]. These curves showed three relevant weight losses. The first thermal change occurred until ~85 °C due to the elimination of absorbed water of the collagen where it also began its denaturation [13,40]. The second significant weight loss occurred ~219 °C due to the decomposition of collagen chains [40,41]. Finally, the most substantial weight loss rate for both samples occurred up to 500 °C, which corresponds to a complete decomposition of both native ECM and dECM-BD [39]. The superposition of the curves shows that there was no alteration of the thermal stability.

The effectiveness of the decellularization process proposed can be attributed to two key factors. First, to the cooperation generated between the reagents used in the decellularization protocol (SDS and DNase-I) and second to the perfusion setting used along the entire process.

Sodium dodecyl sulfate (SDS) is an ionic detergent widely reported in the decellularization of tissues such as the human heart, cornea, and lung [42,43,44,45]. SDS solubilizes the cellular and nuclear membranes with a high index of effectiveness [25]. However, SDS can lead to a higher damage rate of ECM´s collagen fibers [46]. To prevent this, we used an SDS concentration (1% *w*/*v*) previously reported for murine ureter decellularization [9]. To prevent the adverse effects of SDS detergent in our protocol, we extended washing times—about 1/3 of the total protocol time, and we avoided the use of other detergents as triton or sodium deoxycholate as previously reported methods [9,47]. This approach showed that the ECM of the biliary duct was preserved with high fidelity after decellularization when compared to native tissue samples (Figure 2 and Figure 3).

The other main decellularization reagent used in this study was DNase-I, due to its hydrolytic activity on the DNA chain present in the cellular tissue once the cell and nucleus membranes are disrupted following SDS application. The DNase-I is most enzymatically active at 37 °C; however, our preliminary evaluations using this temperature led to degradation of the ECM bile duct. Hence, we decided to carry out the decellularization process at room temperature, preventing dECM-Bd degradation whilst still being effective. DNase-I has been extensively used in decellularization protocols [48,49,50,51,52]. The use of detergents, with or without enzymes, plays a crucial role in the efficiency of decellularization. In this study, more than 97% of DNA content in the native bile duct ECM was removed with a combination of SDS and DNase-I using perfusion decellularization protocol (Figure 2d). Other similar studies related to the obtention and reconstruction of bile ducts with decellularized grafts did not mention the final content of DNA after the decellularization process [9,47]. However, other studies where SDS was used as a decellularization agent could remove more than 90% of remnant DNA [53,54,55,56], which is consistent with our results. This parameter plays a critical role in order to reduce the scaffold’s immunogenicity [7,56].

A critical aspect in all decellularization process is the consuming time to get an effective decellularized matrix. This is important, since long times could lead to degradation of the ECM [39]. Hence, the second key factor in achieving a fast decellularization process was the decellularization circuit designed where the suitable flow rate leads to effective decellularization. Even though the reported normal physiological flow of the bile duct is 1 mL/min [32,57], this flow did not lead to effective decellularization and was increased until complete decellularization of the matrix was obtained in reasonable times (8 days in total) and matrix degradation was reduced to 46 mL/min. Other similar studies have reported around 7 days for complete decellularization of bile grafts. However, this approach has been obtained from different tissue from bile ducts (ureteral grafts) [9]. This suggests that the nature of the decellularization protocol needs to be tailored for distinct organs [39]. Other decellularization studies employing murine bile ducts used detergent-based enzymatic decellularization but failed to report the exposition time. Furthermore, applying murine biliary ducts in human-like models is limited by the inherent properties of murine tissue (e.g., volume and size) [47].

To evaluate the elimination of potential cytotoxic residual in the dECM-BD and to assess the potential scaffold of an acellular matrix as a xenograft, we performed the cytotoxicity test and cell seeding assays (Figure 4 and Figure 5, respectively). We employed PK84 cells, which are a sentinel mesenchymal/fibroblastic cell line that is of human dermal origin. PK84 cells are commonly used in cell-biomaterial contact and cytotoxicity of materials in vitro, with Van Luyn et al. (1991) providing one of their earliest descriptions of their use for biomaterial cytotoxicity assays [58]. Thus, this cell line has been employed for the same purposes as described in our current work for over three decades [59,60]. The decellularized matrix retained the significant features of the native tissue. This provides support and biochemical cues for cellular attachment, proliferation, and migration [7,13,56,61].

Cytocompatibility is one of the main features of success in any biomaterial, and it is closely related to its chemical composition and structure [62]. The biomaterial surface interacts with the cells, promoting cell attachment, proliferation, and infiltration; precisely the last part of this cascade of events was shown by Masson staining after 7 days of cell seeding (Figure 5), which was consistent with similar studies [20]. The present study provides an alternative protocol for decellularization as a possible platform for bile ducts replacement. This study suggests that the essential structural characteristics of the decellularized extracellular matrix of bile ducts were preserved. In addition, the biochemical characteristics of the matrix are beneficial for the reseeded cells to attach them to the dECM-BD remained viable and well preserved. In our future studies, we will focus on preclinical evaluation in animal models of these matrices.

## 5. Conclusions

The decellularization process of bile ducts described in the current work is a fast and effective method that results in the removal of cellular components but that preserves the non-cellular components of the tissue: the ECM. The most important features of the current protocol are that ECM architecture alterations are minimal, and the perivascular lumen is preserved. The potential of regenerating tissues using xenogeneic sources may overcome the current shortage in organ transplantation medicine. Hence, the findings of this study represent an initial approach for bile duct xenograft production. Future studies will assess the cell adhesion of endothelial cells to the vascular plexus of these decellularized matrices and their survival under dynamic conditions. Long-term viability studies may indicate if the dECM-BD scaffold allows not only cell infiltration as demonstrated here but also their proliferation and subsequent survival. These assessments may further characterize the in vitro recellularization of the biliary ducts before in vivo implantation. Other vital tissue properties, such as its innervation, need to be carefully considered for inclusion in the near future.

## Data Availability

All data are presented in the article.
